# The American Ambassador on Hospitals

**Published:** 1906-06-02

**Authors:** 


### THE AMERICAN AMBASSADOR ON HOSPITALS.

### CHARING CROSS HOSPITAL DINNER.

Charing Cross Hospital held its festival dinner on Wednesday, May 23, at the Hôtel Cecil, and as a result collected £12,257. The American Ambassador occupied the chair, and was supported by, amongst others, Lord Kilmorey (Chairman of the Hospital), Lord Weardale, Lord Clifford of Chudleigh, Colonel Sir H. Hozier, Sir J. Heron Maxwell, Bart., Sir C. Morrison Bell, Sir Richard Awdry, Sir Edward Stern, the Hon. J. G. Jenkins, Count Ward, Lieut.-Colonel Clifford Probyn, Messrs. Stuart M. Samuel, M.P., T. P. Borrett (Vice-Chairman of the Hospital), Charles Drummond, H. A. Harben, and Henry Wagner.

During the year 1905 2,465 in-patients (a number exceeding that of any previous year), and 20,552 out-patients were treated at the hospital. Of the last-mentioned figure 10,385 were cases of accident and emergency.

The loyal toasts having been honoured,

The American Ambassador proposed "Prosperity to the Hospital." He said :—

I rise now, an American at this English board, to propose the special toast of the evening—prosperity to a typical London hospital. If anyone should think that this aggregation of circumstances is somewhat anomalous the explanation is simple. One of the very active workers for this hospital has been unable to continue her usual activities in its behalf. For two years she has been suffering from a tragic calamity which she has borne with the courage of a hero and the patience of a martyr. In the agony of her constant suffering, her thoughts still turned to this hospital, and she called on one and another for tasks which she thought might, in some small degree at least, help to supply the lack of work which her absence had entailed. One at least of her friends, and an American, was proud to respond to the pathetic appeal from a chamber of suffering and to do whatever he could, however small it might be, to further her wishes in behalf of the thousands of other sufferers to whom her sympathy went out. (Hear, hear.)

#### Our Duty to the Hospitals.

It is certainly a marked distinction of this Metropolis, this world, this London, that amid all your multifarious activities in behalf of human progress, in your efforts to lift up and develop and forward humanity, the most conspicuous, perhaps, of all your benevolent efforts, is your work in behalf of hospitals. It is perhaps the only species of benevolent effort to which the complaint cannot be addressed that it may sometimes extend aid where at the most only "help to help themselves" ought to be given. But in the case of the hospitals, there is no question of self-help, no question of the immediate need and no question of the immediate duty. So long as the highest possible duty rests upon the fortunate to help the unfortunate in time of suffering—and I take it that most of us will assent to the proposition that that is the plainest and simplest duty of ordinary humanity—so long as that remains it will be true that the surest and highest way to do good is to support the hospitals. (Applause.) It is certainly also the safest way, at least for busy people. No man has the right to give indiscriminately to applicants without investigation. (Hear, hear.) That is the surest way in many cases to pauperise and demoralise the recipients. Yet for the busy man who cannot investigate, how else can he do his duty and give aid so surely as by giving that aid, in cases where there is no possibility of question, for present relief of actual and known suffering. The one case in which it is possible to make no mistake about charity is to give present relief to present suffering, and that is a duty which rests alike upon all of us in proportion to our means and in accordance with our situation. (Applause.)

### The Voluntary Hospital System.

It has always seemed to me a very beautiful trait of the English character—perhaps I may extend that remark and say that it is a very beautiful trait in the character of all English-speaking people—(hear, hear)—first that they maintain such magnificent hospital systems, and then that they maintain them largely by voluntary effort. (Hear, hear.) Elsewhere the hospital is apt to be a Government affair, almost universally, I think. On the contrary, among our Anglo-Saxon communities, following, I think, the Anglo-Saxon instinct and the really human, manly impulse, it is a direct thing between the man who is able to give and the man who needs. As such, it seems to me the link which binds all classes together—(applause)—but somehow of late, in other cities as well as London, and in other countries besides Great Britain, there seems at times to be something wrong with the hospitals, something is not going right. They are apt to be poor. This hospital, in spite of the magnificent support it has received, in spite of the very generous contributions, the very admirable list of patrons, and the enormous amount of good work that has been given to it, this hospital I am told on authority is poor.

### Why our Hospitals are not Properly Supported.

Well, there are two reasons generally assigned for this poverty of hospitals, and I assume that they apply to the Charing Cross Hospital. The first reason is that hospitals are often called upon to support and care for patients who really could provide, or whose friends could provide, for their support elsewhere, at institutions not open and free to the general public. If that is the cause here it would seem to be a matter, somewhat at least, within your own control. The other reason seems to me to be more serious. There has been heard, not only in London, but in other large cities in other countries, a complaint that the support of the hospitals is not what the increasing demands of the times require, and that they do not receive support proportionately from so large a class as formerly. In fact, I have heard it stated, on what I suppose to be very high authority, with reference to this City of London, that not more than one in ten of the well-to-do householders is a contributor to the hospitals' funds. I do not vouch for that fact, for I could not personally know whether it is correct or not, but I have been told it on what I suppose to be excellent English authority. If that is a fact it seems to me to be a very lamentable fact. Surely one-tenth of the capable community ought not to support the burden which belongs just as much to the other nine-tenths. (Hear, hear.) We cannot even tell for how long the one-tenth may feel that they can continue to bear that burden, but, at any rate, it is certainly the duty and the interest of the other nine-tenths to bear their full share in helping to keep the hospitals of this great Metropolis fully up to date and up to the demands of a growing population and an increasing activity which forces more work than ever upon the hospitals. (Hear, hear.)

### Our Duty and Interest to Support Hospitals.

I said it was their duty and their interest. Perhaps it has not been pressed home as much in the past as it might be, that it is the interest of every well-to-do citizen to see that the hospital system of this City is in splendid condition, fully up to the times and well supported. It is his interest because otherwise he has no security for the safety of himself, his wife, and his children. We all know what progress has been made in medical science in the last half century, in the last quarter of a century, but do we, as often as we should, reflect upon the fact that that progress rests on the assistance given to the medical profession by the hospitals? It is to his interest, too, for another reason besides this. If the hospitals are not supported, if the one-tenth grow weary of bearing the burden of the other nine-tenths, what follows? Merely that the hospitals run down, and that the men who have neglected their duty will be haunted not only in their consciences, but on their very doors and in the streets by the sick whom they have neglected to provide for. You would recur to the condition of the bad old medieval times. That would seem to make it fairly clear that it is to the interest of the other nine-tenths to support the Charing Cross Hospital and every other hospital in the City of London which deserves it as well. (Applause.) But it is also their duty because no one can escape the Divine command "Minister to the afflicted"; their duty, because otherwise they would sever that tender tie which binds those of us who have means to those who have not by the sympathy and kindness of aid given when it is needed, a tie which springs out of our common humanity, out of the brotherhood of the race, and its common fatherhood. (Applause.) If you sever that tie between the well-to-do and those who are not well-to-do there yawns before us a chasm none of us wish to look into. In any case Charing Cross Hospital is here and must stay here, and it must be supported. If the general hospital support is not kept up to the demands of the times I think there is danger: the danger that if the nine-tenths will not do their duty then the one-tenth may stop and we shall have to call upon the law to extort from everybody the support which has to be given to the inmates of our hospitals. (Applause.) But let us hold on, if we can, to the direct, natural, Anglo-Saxon way of dealing with cases of suffering, straight from the well-to-do to those who are in distress, without the intervention of the law and by the action solely of the natural promptings of humanity. (Applause.)

Lord Kilmorey, in responding, explained that he was putting into operation a scheme of house-to-house collection in the district which the Charing Cross Hospital serves, based on the plan adopted by the Edinburgh Royal Infirmary. From this he hoped for great results which might possibly put the hospital finances on a firm basis.

